# Durable viral suppression in the dolutegravir era: a data-enabled, risk-stratified model for integrated HIV care in Uganda

**DOI:** 10.3389/fpubh.2026.1794681

**Published:** 2026-05-04

**Authors:** Maria Magdalene Namaganda, Joyce Nakatumba Nabende, David Patrick Kateete, Charles Batte, Gerald Mboowa

**Affiliations:** 1 Department of Immunology and Molecular Biology, School of Biomedical Sciences, College of Health Sciences, Makerere University, Kampala, Uganda; 2 Department of Computer Science, School of Computing and Information Technology, Makerere University, Kampala, Uganda; 3 Lung Institute, School of Medicine, College of Health Sciences, Makerere University, Kampala, Uganda; 4 African Center of Excellence in Bioinformatics and Data-Intensive Sciences, Makerere University, Kampala, Uganda

**Keywords:** differentiated service delivery, dolutegravir, East Africa, electronic health records, health equity, HIV, non-communicable diseases, tuberculosis

## Abstract

Despite widespread rollout of dolutegravir-based (DTG) antiretroviral therapy (ART) in East Africa, viral non-suppression, advanced HIV disease (AHD), and multimorbidity persist, reflecting gaps in service response rather than regimen potency alone. In Uganda’s The AIDS Support Organization (TASO) routine-care cohort (2014–2024; *n* = 54,348 people living with HIV), integrase inhibitor uptake is near universal, yet AHD remains common, and tuberculosis (TB) and non-communicable diseases (NCD) increasingly co-occur within HIV care. Among clients with a recorded most recent viral load, 6.4% (2,145/33,384) had viral non-suppression (VL ≥ 1,000 copies/mL). Second, our regional systematic review and meta-analysis (2016–2023; *n* = 29,829) estimates viral non-suppression at 19.4% and indicates that failure concentrates in predictable social and clinical risk strata. We propose that durable suppression may be strengthened by an accountable, time-bound viral load (VL) cascade, paired with targeted support for clients at elevated clinical and social risk. Building on WHO and national differentiated service delivery and AHD guidance, we outline a pragmatic, data-enabled, risk-stratified model that uses routine electronic health record (EHR) signals to trigger rapid viral non-suppression follow-up, guide delivery of a proposed time-limited adherence and socioeconomic stability bundle, and integrate TB and NCD management within HIV platforms. A minimum actionable dashboard focused on cascade timeliness, high-risk package delivery, and integrated care can translate ART scale-up into durable suppression, fewer preventable AHD complications, and faster progress toward 95–95–95.

## Highlights

- Viral non-suppression; elevated viral load (VL): HIV RNA ≥ 1,000 copies/mL (programmatic threshold), prompting time bound follow-up and adherence support.
- Durable viral suppression: two consecutive VL results <1,000 copies/mL at least 6 months apart, with no recorded VL ≥ 1,000 copies/mL in the preceding 12 months (where multiple VL measures are available).
- Confirmed virological failure (VF): repeat VL ≥ 1,000 copies/mL after completed enhanced adherence counselling (EAC), requiring clinical review and regimen optimisation per national guidance. EAC may be termed intensive adherence counselling (IAC) in some settings, we use EAC throughout.
- Advanced HIV disease: CD4 < 200 cells/μL and/or WHO stage III/IV (or national definition); used here as a marker of elevated morbidity and mortality risk even in mature ART programmes.
- Risk tiering: a rules-based triage from routine EHR variables to trigger service intensity (not to replace clinician judgement or to label clients).

## 1 Why viral non-suppression persists in the integrase inhibitor era

*Why now*: As antiretroviral therapy (ART) coverage and DTG-based regimens become the norm for people living with HIV, remaining viraemia increasingly reflects VL cascade delays, missed visits, social vulnerability, and comorbidity not pharmacologic potency alone. A shift from ‘coverage’ to ‘durability’ requires measurable, time-bound clinical pathways and equity-aware differentiated service delivery (DSD) (1). Importantly, the proposed risk-stratified model is designed to support, not replace, clinical decision-making. Risk tiering and EHR-enabled alerts function as prompts for structured clinical review, while final decisions remain under the authority of trained healthcare providers.

Over the past decade, Uganda and its Eastern African neighbors have delivered one of the most consequential public health gains of the modern era: rapid expansion of ART, wider access to laboratory monitoring, and guideline shifts toward earlier treatment initiation and more potent regimens. However, as programs mature, success requires both high viral load coverage and durable suppression. As coverage expands, marginal gains increasingly depend on sustaining suppression over time and acting promptly on elevated results. Persistent virological failure (VF) or non-suppression undermines individual health, increases the risk of onward transmission, and creates selective pressure for antiretroviral drug resistance (2). Importantly, evidence from African settings shows that delays in acting on confirmed virological failure are common and clinically significant. Such delays may prolong periods of viraemia, reflect modifiable programmatic gaps, and are associated with worse outcomes, including increased mortality among patients with low CD4 counts at failure (3, 4). In this context, the central policy question is no longer primarily *“Which regimen?”* but rather: “Which package of services, for which clients, at what point in the care trajectory, delivered with what accountability and timeliness?”

Two complementary evidence streams highlight this transition. First, Uganda’s national routine-care cohort from The AIDS Support Organization (TASO) followed at least 54,348 people living with HIV across 11 clinics from 2014 to 2024, capturing a decade of program evolution under real-world delivery conditions (5).

We document rapid programmatic transition from predominantly non-nucleoside reverse transcriptase inhibitor (NNRTI) regimens to widespread Integrase Strand Transfer Inhibitor (INSTI)-based therapy following the dolutegravir (DTG) rollout. However, viral non-suppression persists among clients on ART and those re-engaging in care, alongside ongoing AHD risk, rising multimorbidity and socioeconomic vulnerability. Among clients with a recorded most recent viral load, 6.4% (2,145/33,384) had viral non-suppression (VL ≥ 1,000 copies/mL); at initial VL, 8.6% (2,361/27,599) were non-suppressed (6). Second, our regional systematic review and meta-analysis covering 2016–2023 (*n* = 29,829) estimates pooled East African VF at 19.4% and shows that VF clusters in predictable risk strata driven by socioeconomic vulnerability (such as poverty/income insecurity, transport barriers, stigma, competing demands); disease severity, and adherence-related barriers (7). Together, these data suggest that VF in the current era is increasingly a predictable program outcome and therefore preventable if policy and service design align with risk.

## 2 What the Uganda TASO cohort reveals about a maturing ART program

Evidence base (TASO cohort): TASO maintains a national routine-care registry across 11 centres of excellence in Uganda. The cohort is an open, longitudinal routine-care cohort of 54,348 people living with HIV (PLWH) receiving care between 2014 and 2024, with routine capture of demographics, ART regimen history, viral load results, CD4/WHO stage where available, TB history, selected NCD diagnoses, and socioeconomic/psychosocial indicators. We analyzed records of PLWH receiving care at TASO-Uganda clinics from 2014–2024 to characterize their demographic, clinical and treatment profiles; and interpret patterns at baseline (earliest recorded value) and follow-up (most recent recorded value), recognizing variable completeness typical of routine data (8). In this Perspective, we use the TASO cohort to characterize the programmatic, clinical, and social context in which durable viral suppression must be achieved, including regimen transition, residual advanced HIV disease, multimorbidity, and structural vulnerability that may shape care complexity and the risk of delayed response to viral non-suppression. Several patterns are particularly salient for a forward-looking HIV policy agenda. First, regimen transition has been swift. At ART initiation, NNRTI-based regimens dominated through 2017, and by 2024 over 99% of clients were on INSTI-based regimens. At the most recent recorded regimen, 97% of clients were documented as receiving first-line ART; this reflects routine-care regimen classification at the most recent visit and should not be interpreted as uninterrupted first-line treatment success across follow-up.

Second, earlier HIV diagnosis and immune recovery have improved, but advanced disease has not disappeared. At ART initiation, 12% of clients had CD4 < 200 cells/μL. Among those with a recorded most recent CD4 result, 18% remained below 200 cells/μL, while 46% had CD4 > 500 cells/μL. We interpret this as residual severe immunosuppression in a subset of clients; however, this should not be equated with ongoing virological failure, as incomplete immune recovery may persist despite virological suppression. In routine care, this subgroup remains clinically important because low CD4 may still signal elevated morbidity risk and greater service complexity.

Third, comorbidity is now a central feature of HIV care in Uganda (6, 9). TB remains the most frequent infectious comorbidity (5% prior TB history recorded), and NCDs are increasingly visible in the routine data: hypertension (3.9%) and diabetes (2.5%) were the leading documented chronic conditions. These figures likely underestimate true prevalence due to incomplete screening and documentation, but they nonetheless signal the epidemiologic transition faced by maturing ART cohorts.

Fourth, the cohort reveals structural vulnerability at scale. Employment data show widespread income insecurity, 57% reported irregular income and 21% were unemployed at ART initiation. Psychosocial assessments identified poverty as the most common recorded stressor (34%), alongside stigma (1.8%) and depression (8.7% among those with screening documented). These attributes are not peripheral; they shape the probability of missed visits, treatment interruptions, and delayed response to viral non-suppression (5).

In aggregate, the TASO cohort argues for a policy shift: from a ‘one-size-fits-most’ model optimized for regimen distribution to a data-enabled differentiated system optimized for durable suppression among heterogeneous risk groups, while recognizing that direct quantification of care pathway delays will require dedicated longitudinal cascade analyses.

## 3 Regional evidence: virological failure is common and socially patterned

Evidence from the systematic review with meta-analysis and meta-regression: By systematically evaluating and synthesizing evidence from published studies between 2016 and 2023, this review sought to; estimate the pooled prevalence of VF across East African countries, identify and quantify the impact of key demographic, clinical, and programmatic factors associated with VF in this region, explore potential sources of heterogeneity across studies through meta-regression analyses and highlight knowledge gaps and areas requiring further research to inform evidence-based strategies for improving viral load monitoring and enhancing treatment outcomes. The East African systematic review and meta-analysis provides an essential regional context. Across 25 studies from 2016 to 2023 (total *n* = 29,829), pooled VF prevalence was 19.4% (95% CI 15.2–24.0), with substantial heterogeneity, a reminder that VF levels are sensitive to the performance of local healthcare systems (distance to healthcare facilities, laboratory networks, result return, adherence support, and clinical decision-making) and to the mix of risk strata in each setting. Importantly, VF concentrated in identifiable and reproducible strata (7).

Sociodemographic predictors were consistently associated with higher VF prevalence, including male sex (30.9%), unmarried status (28.2%), lower educational attainment (33.0%), non-formal employment (47.2%), and urban residence (51.2%). Clinical predictors included low CD4 count (35.1%), advanced WHO HIV/AIDS stage III/IV (44.2%), HIV/TB co-infection (24.3%), and other opportunistic infections. Poor adherence was a strong proximal pathway (41.8% VF among those categorized as poor adherence), and some first-line regimen anchors (e.g., nevirapine-based regimens) were associated with higher VF in historical cohorts (7).

This pattern has two policy implications. First, VF may be partially predictable using variables already captured in routine care data, supporting the rationale for simple, transparent risk stratification approaches that require prospective validation. Second, VF reflects persistent inequities: structural barriers and social determinants continue to shape adherence and retention even as treatment options become more potent and convenient. Importantly, residual VF can persist despite widespread availability of high-barrier INSTI-based therapy (9, 10) and even next-generation modalities such as long-acting injectable ART (e.g., cabotegravir–rilpivirine) (11); therefore, policies that interpret VF primarily as pharmacologic failure risk overlooking the most actionable causes such as missed visits, delayed VL action, economic insecurity, stigma, and fragmented TB/NCD care. Programmes should couple modern regimens with accountable, time-bound VL cascade action and targeted socioeconomic and integrated clinical support. We note that while these patterns are consistent across regional evidence, the current TASO analysis is descriptive and does not directly estimate time-to-resolution of viral non-suppression or causal effects of individual risk factors, which will require dedicated longitudinal analyses.

## 4 A policy and service design agenda for the future of HIV care

This section translates the TASO cohort and regional meta-analysis into a pragmatic service design agenda for durable viral suppression in Uganda and similar settings. We propose a set of implementable policy levers that: (i) treat the viral load cascade as an accountable, time-bound care pathway; (ii) embed adherence support within socioeconomic and mental health–informed services for high-risk clients; (iii) integrate TB and NCD care within HIV platforms; and (iv) institutionalize routine EHR-enabled risk tiering to trigger timely intensification and de-escalation of care. Together, these actions shift programs from measuring VL coverage to ensuring VL action. We emphasize that AHD, TB, and NCD integration are presented here as markers of care complexity and implementation priorities for higher-risk clients, rather than as independently validated causal determinants of delayed VLNS resolution in the current TASO analysis.

### 4.1 Make the viral load cascade a time-bound “care pathway,” not a test

Many programmes have expanded viral load (VL) coverage (12), but the cascade from specimen collection to clinical action remains fragile in many settings (3, 4, 13–17). Policies should specify algorithm-aligned timelines after an elevated VL: (i) result return to clinic/client; (ii) initiation of EAC within 14 days of result receipt; (iii) completion of ≥3 EAC sessions spaced ~1 month apart; (iv) repeat VL collected ~1 month after the 3rd EAC session; and (v) clinical review and regimen decision-making within 2–4 weeks of confirmed virological failure, that is, repeat VL ≥ 1,000 copies/mL, per national guidance (12). These steps require operational accountability, routine dashboards that track turnaround time (draw → result → action), supervision, and clinical mentorship to avoid both harmful delays in switching when indicated and premature switching when adherence barriers are remediable. Observational evidence from southern Africa shows that delays in switching after confirmed virological failure are common, are partly driven by modifiable programmatic gaps, and may be associated with worse clinical outcomes, including higher mortality, particularly among patients with low CD4 counts at failure (3, 4).

### 4.2 Embed adherence within socioeconomic protection and mental health-informed services

In the TASO cohort, poverty and income insecurity are common, and the meta-analysis shows that non-formal employment and other markers of disadvantage are associated with higher virological failure (5, 7). These findings support the need for more targeted follow-up for clients facing predictable social and structural barriers, while recognizing that the effectiveness of specific support models should be prospectively evaluated. In this context, potential implementation options may include transport assistance, nutrition or food-support linkage for clients with food insecurity, targeted case management for those with repeated missed visits, and stigma- and mental health-informed services that recognize depression, non-disclosure, and related psychosocial stressors as potential threats to adherence. Rather than universally applied add-ons, such supports may be most appropriate as targeted, time-limited components of a higher-intensity follow-up package for clients with elevated viral load and documented vulnerability, aligned with programme resources, sustainability, and subsequent viral load outcomes.

### 4.3 Integrate TB and NCD services into HIV platforms to reduce fragmentation

A maturing ART cohort experiences multimorbidity (14) Fragmented vertical services create missed opportunities for screening and treatment, increase visit burden, and reduce retention. Policy should prioritize integrated TB and NCD care within HIV Differentiated Service Delivery (DSD) models, including symptom screening and diagnostics for TB, blood pressure measurement and diabetes screening, and simplified referral pathways for complications. Integration is not only a clinical quality issue; it is an adherence and retention strategy.

### 4.4 Institutionalize routine EHR-enabled risk prediction for proactive intensification

Our studies suggest that high-risk clients can be identified using variables already present in routine EHR: demographics, baseline and follow-up clinical status (WHO stage/CD4 where available), TB history, prior elevated VL, visit/refill patterns, and socioeconomic indicators (7).

Programmes should prioritize a simple, transparent risk scoring approach (or rules-based triage) that can be implemented within existing EHR platforms and audited for fairness. The purpose is not to replace clinical judgement or to label clients, but to operationalize proactive service intensification: more frequent contact, targeted adherence support, expedited clinical review, and integrated comorbidity care for those most likely to experience viral non-suppression. These levers are synergistic: a time-bound VL pathway is more effective when embedded within a system that can identify risk early, address structural barriers, and deliver integrated care.

Predictive model development and validation are being pursued separately and will be reported as a dedicated methods paper (*Namaganda, unpublished data*). In practice, this approach enables proactive rather than reactive care. For example, a client flagged as high risk due to a recent elevated viral load and missed visit would trigger immediate follow-up, including rapid result communication, targeted adherence support, and expedited clinical review, thereby reducing delays that commonly lead to virological failure.

### 4.5 From advanced HIV disease identification to durable viral suppression: an accountable, time-bound care pathway

We conceptualize viral non-suppression and preventable morbidity as part of a continuous pathway linking AHD to long-term treatment outcomes, rather than as isolated clinical failures. In this framework, risk stratification is the entry point, using routinely available EHR signals including CD4 count <200 cells/μL where available, WHO stage III/IV events, prior viral non-suppression, recent treatment interruption, TB flags, and socioeconomic vulnerability to explicitly identify individuals at elevated risk of AHD-related morbidity and mortality. This approach acknowledges that AHD persists in routine care even in the era of potent integrase inhibitor–based regimens and that timely identification is essential to prevent avoidable deaths.

Clients classified as high risk trigger a structured, time-bound care response that integrates three elements: (i) completion of the AHD package, (ii) an accelerated viral load cascade, and (iii) integrated TB and comorbidity management. The AHD package is operationalized in line with World Health Organization guidance and national policies, emphasizing rapid TB symptom screening and diagnostic completion, screening and treatment of key opportunistic infections where indicated, initiation of appropriate prophylaxis, and intensified clinical follow-up. Rather than introducing new clinical protocols, our model focuses on ensuring that these evidence-based interventions are delivered on time, consistently, and to those most likely to benefit.

The viral load cascade from specimen collection to result return and documented clinical action is treated as a core accountability pathway within AHD management. Elevated VLs automatically trigger predefined response timelines, including rapid result communication, regimen or adherence review, and follow-up testing, thereby reducing delays that disproportionately harm clients with advanced disease. This emphasis on timeliness is supported by prior African evidence showing that prolonged delays in switching after treatment failure can worsen outcomes, with the greatest risk observed among those with advanced immunosuppression at failure (3, 4). This VL-focused accountability may be complemented by a targeted, time-limited adherence and socioeconomic support package for high-risk clients, particularly where structural barriers such as food insecurity, transport costs, stigma, and mental health challenges are documented and where programme resources permit.

To support routine implementation and monitoring, we extend our proposed minimum actionable dashboard to include AHD-specific indicators that make care gaps visible and actionable. These include: (1) the proportion of newly enrolled or re-engaging clients assessed for AHD risk within a defined timeframe; (2) the proportion of identified AHD clients completing TB and NCD screening and diagnostic pathways within target timelines; (3) the proportion of AHD clients initiated on appropriate prophylaxis according to national guidance; and (4) short-term (30- and 90-day) outcomes for AHD clients, including retention in care, mortality where available, and timeliness of VL action following an elevated result. Together, these indicators shift monitoring from retrospective reporting to real-time service accountability.

By explicitly linking AHD identification to a time-bound, integrated care response and embedding accountability within routine EHR systems, this pathway operationalizes the WHO AHD package in everyday programme settings. In doing so, it reframes AHD not as a failure of treatment potency, but as a systems and equity challenge that can be addressed through deliberate design of data-triggered actions that bridge the gap between guidelines and practice, ultimately enabling durable viral suppression and reducing preventable morbidity and mortality in sub-Saharan Africa.

Implementation of this approach is feasible within existing clinic workflows in resource-constrained settings especially in Africa. Nurses and clinicians can apply AHD risk tiering at enrolment or re-engagement using routinely available data (recent viral load, clinical stage, treatment interruption), while community health workers support follow-up, adherence, and socioeconomic stabilization for high-risk clients. Data clerks or existing health information staff can configure simple EHR alerts and dashboards to flag overdue actions without requiring new platforms. By distributing tasks across cadres already embedded in national HIV service delivery, the model emphasizes coordination and accountability rather than additional staffing or parallel systems.

## 5 Operational blueprint

**Figure 1:**
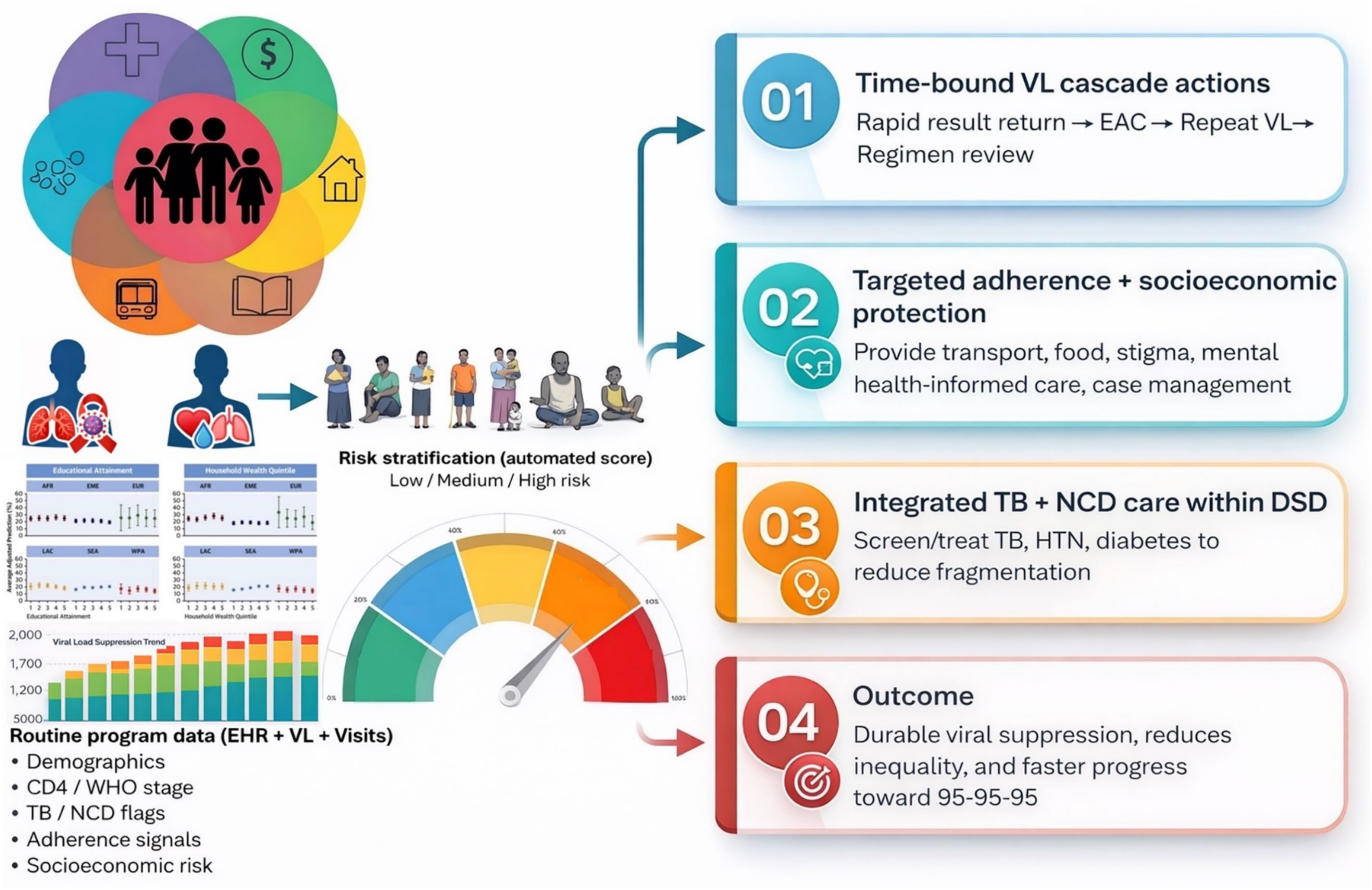
A data-enabled, risk-stratified differentiated service delivery (DSD) model to prevent viral non-suppression. Routinely collected program data (electronic health records, VL results, and visit history) including demographic, clinical (CD4/WHO stage), TB/NCD indicators, adherence signals, and socioeconomic risk markers are used to generate a rules-based (or automated) risk tier (low/medium/high). High-risk = elevated VL (≥1,000), plus ≥1 risk flag (missed visit/refill gap >14 days; AHD marker CD4 < 200/WHO III–IV where available; TB/OI flag; prior elevated VL; or documented high vulnerability). *Minimum escalation trigger*: elevated VL + one additional risk flag (with clinician override). Where dashboards are not feasible, the same triggers can be implemented using paper line lists and phone-based follow-up, with monthly facility review of draw → result → action performance. Risk tiering then triggers a coordinated package of interventions: (i) Time-bound viral load cascade actions (rapid result return, enhanced adherence counseling, repeat VL, and timely regimen review), (ii) Targeted adherence and socioeconomic support (e.g., transport/food support, stigma and mental health–informed care, case management), and (iii) Integrated TB and NCD care within DSD (screening and treatment pathways for TB, hypertension, and diabetes). The intended outcome is durable viral suppression, reduced inequities, and accelerated progress toward 95–95–95 (18).

Figure 1 presents a pragmatic implementation model, and Table 1 links each policy lever to specific actions and a minimum indicator set for routine performance management and equity monitoring. The model begins with routinely collected EHR data that are already available in many programs such as clients’ demographics, WHO stage or CD4 (where available), NCDs/TB history, visit/refill patterns, prior elevated VL, and basic socioeconomic flags. These inputs feed a transparent score (or ruleset) that assigns clients to low-, medium-, or high-risk tiers, updated at each VL result or key clinical encounter. Risk tiering then activates predefined service “packages”: low risk supports streamlined differentiated service delivery (e.g., multi-month dispensing and fewer clinic visits); medium risk adds structured adherence check-ins plus integrated TB/NCD screening; and high risk may trigger a targeted, time-limited intensification package that combines time-bound VL cascade actions with case management and selected socioeconomic support, depending on documented need, local feasibility, and available programme resources. Implementation can start with a minimum viable dashboard that tracks; draw → result → action time, timeliness of enhanced adherence counseling (EAC) initiation, repeat VL completion, re-suppression, retention, and equity gaps by sex, age, site, and available vulnerability markers. Throughout, confidentiality safeguards and clinician co-design are essential to ensure risk stratification is used for support not punitive performance management.

**Table 1:** Compact policy-to-implementation mapping for AHD and durable viral suppression.

Policy lever	Implementation actions	Suggested indicators (minimum set)
(1) Time-bound viral load (VL) cascade + AHD risk-triggered intensification	Define explicit, operational timelines after an elevated VL (e.g., result returned to clinic within 7 days of availability; enhanced adherence/implementation counselling initiated within 14 days of result receipt; repeat VL per national algorithm; clinical review for confirmed virological failure within 2–4 weeks). Embed EHR-based risk tiering (including AHD where available) to trigger intensified follow-up, assign accountability (facility owner + lab focal point), and use monthly dashboard review + clinical mentorship to close gaps. Include de-escalation criteria once re-suppressed and visit-stable.	VL cascade timeliness: median days specimen draw → result available; median days result available → client notified/seen; median days elevated VL → documented action. Coverage: % elevated VL results received at clinic within 14 days of draw; % elevated VL clients contacted/seen within 14 days of result. Response: % elevated VL with counselling started within 14 days; % completing counselling sequence; % repeat VL completed within guideline window; % re-suppressed within 6 months of index elevated VL. AHD risk assessment: % newly enrolled or re-engaging clients assessed for AHD risk within 7 days of enrolment/re-engagement (CD4 < 200 cells/μL or WHO stage III/IV where available). High-risk action: median days confirmed repeat VL ≥ 1,000 copies/mL → regimen decision/clinical action; disaggregate by AHD and vulnerability markers.
(2) Targeted socioeconomic + adherence support for high-risk clients (including AHD)	For high-risk clients, consider a targeted, time-limited follow-up package with case management and selected socioeconomic or psychosocial supports where documented barriers are present and local resources permit. This package is proposed as an implementation-focused, hypothesis-generating approach and should be prospectively evaluated.	Missed-visit/late attendance (>7/14/28 days) and pharmacy refill gaps among high-risk clients. Retention at 6 and 12 months (overall and high-risk/AHD strata). % high-risk clients receiving ≥2 bundle components within 30 days of risk flag; % receiving documented follow-up contact within 7 days of missed appointment. Re-suppression after elevated VL among high-risk clients; time to re-suppression (median days).
(3) Integrated TB + NCD care within HIV differentiated service delivery (DSD), with AHD package delivery	Integrate TB and NCD screening/management into HIV refill/visit platforms (including community models): TB symptom screen and diagnostic completion; TB preventive therapy (TPT) initiation where eligible; blood pressure and diabetes screening; comorbidity registers; referral loops avoid parallel queues. For AHD clients, prioritize rapid TB evaluation and opportunistic infection (OI) screening/treatment and prophylaxis per national guidance.	TB screening: % clients with TB symptom screen documented at last visit; % symptomatic clients completing TB evaluation within 14 days. AHD-TB completion: % AHD clients completing TB diagnostic pathway within 7 days of AHD identification (or per national targets). Prophylaxis/OI package: % AHD clients started on appropriate prophylaxis per national guidance within 7 days of identification (e.g., cotrimoxazole; TPT where eligible; cryptococcal screening/therapy where applicable and available). Comorbidity integration: hypertension and diabetes screening coverage; % with controlled BP/glucose among those diagnosed; % DSD visits with integrated TB/NCD services.
(4) Data governance + dashboard accountability (including AHD indicators)	Implement a minimum viable dashboard (site + district + national views) with role-based access, audit logs, and privacy protections. Review monthly in continuous quality improvement (CQI) forums and use findings to deploy targeted support (lab–clinic interface fixes, mentorship, supply chain). Ensure AHD fields and cascade timestamps are captured reliably.	% sites submitting complete data on time; documented monthly dashboard review and action log. Completeness of key fields: VL dates/results, visit/refill dates, counselling milestones, TB screening outcomes, and AHD risk fields (CD4/WHO stage where available). Equity gaps: draw → result → action timeliness, AHD package completion proxies, and suppression outcomes disaggregated by sex, age, site, and vulnerability markers. Short-term outcomes for AHD clients: 30- and 90-day retention; mortality where available; and VL action timeliness following elevated VL.

The table links policy levers to implementable actions and a minimum indicator set for routine performance management across the AHD-to-suppression pathway, including AHD risk identification, timely delivery of intensified packages, and an accountable VL cascade from specimen collection to clinical action. “X” should be defined by national guidance or local SOPs (e.g., days to AHD risk assessment and package completion, days to VL result return, timing of enhanced adherence counseling (EAC) initiation, and repeat VL interval). Indicators should be reported overall and stratified by sex, age, site, and available socioeconomic risk markers (and, where feasible, baseline CD4/WHO stage) to monitor equity and identify subgroups at highest risk of preventable morbidity and mortality. AHD package includes TB plus cryptococcal disease screening where diagnostics are available, prompt management of severe bacterial infections, PJP prophylaxis where indicated, and nutritional assessment/support, alongside cotrimoxazole and other prophylaxis per national guidance.

AHD, advanced HIV disease; VL, viral load; EAC, enhanced adherence counseling; DSD, differentiated service delivery; TB, tuberculosis; NCD, non-communicable disease; EHR, electronic health record; OI, opportunistic infection.

Worked example (minimum viable pathway): A client with VL ≥ 1,000 copies/mL triggers ‘high-risk’ follow-up if they also have a recent missed visit/refill gap (>14 days) and/or advanced HIV disease (CD4 < 200 and/or WHO stage III/IV). Within 7 days, results are returned and EAC is initiated promptly (target ≤14 days of result receipt) and delivered as ≥3 sessions spaced about 1 month apart. If repeat VL remains ≥1,000 copies/mL, conduct clinical review and regimen decision-making within 2–4 weeks of the repeat result (per national guidance). If repeat VL re-suppresses, de-escalate to streamlined DSD once visit stability is restored for about 6 months. For instance, in a typical clinic setting, a nurse or clinician reviewing the daily dashboard may identify a high-risk client flagged for delayed viral load follow-up. This would prompt immediate verification of result receipt, initiation of enhanced adherence counselling, and coordination with a case manager or community health worker for patient tracing and support. Such workflows demonstrate how routine data can be translated into timely, team-based clinical action without introducing additional system complexity.

The model can be implemented at three levels of digital maturity. (1) *Paper-first clinics*: use a weekly paper line list of clients with elevated VL, overdue repeat VL, or missed visits; phone/text follow-up; and a simple log to track draw → result → action milestones. (2) *Basic EHR clinics*: use simple flags and worklists within existing registers/EHR and review in weekly clinical huddles. (3) *Dashboards*: where data staff exist, use a simple dashboard to strengthen accountability and track pathway timeliness and equity.

The pathway is anchored to routine touchpoints: enrolment/re-engagement triage, refill encounters, receipt of VL results, counselling visits, and missed-visit tracing. Responsibilities are assigned to clinician/nurse for clinical action; counsellor/CHW for follow-up; facility lead/lab focal point for monthly review.

This workflow aligns directly with the World Health Organization AHD package, which emphasizes systematic identification of AHD, intensified TB case finding, screening and treatment of key opportunistic infections (including cryptococcal disease where diagnostics are available), provision of prophylaxis per national guidelines, and closer clinical follow-up. While these components are well defined in policy, their timely and complete delivery in routine care remains inconsistent. Our contribution is to operationalize the AHD package as a time-bound, accountable cascade, using routine EHR signals to trigger specific actions and track completion, thereby closing the gap between guideline intent and real-world implementation.

The minimum actionable dashboard serves as the operational interface linking data to practice, ensuring that identified risks translate into accountable clinical and programmatic responses across all levels of care.

## 6 Workforce readiness and organizational adoption

Risk tiering and dashboard alerts support clinical decision-making, not replace it. When an EHR flag identifies a high-risk client, the system prompts a structured review; the clinician determines the response. Provider training should focus on interpreting risk outputs in context and using the VL cascade as a workflow guide, not an automated protocol. Social workers, case managers, and community health workers are central to translating these outputs into action. These cadres must be trained to respond to specific EHR triggers such as a flagged missed visit, an overdue VL result with targeted follow-up including transport coordination, nutrition linkage, mental health referral, and adherence support. To ensure safe and effective use, training should explicitly emphasize that risk scores are decision-support tools rather than prescriptive algorithms. Clinical judgement remains essential, particularly in complex cases where social, behavioral, and comorbidity factors may not be fully captured in routine data systems.

High-risk clients with advanced disease, multimorbidity, and compounding socioeconomic barriers require more complex case management and more provider time than stable clients on streamlined DSD. Staff managing high-risk caseloads should receive focused training in integrated TB/NCD assessment and mental health-informed adherence counselling, and caseload allocation should account for the additional time these clients require. In practice, this implies differentiated workload allocation, where providers managing high-risk clients are supported with reduced caseloads or additional multidisciplinary support to ensure adequate time for comprehensive care delivery.

Facilities adopting this model should assess readiness before implementation: provider capacity to use dashboards, availability of trained case management and community health worker cadres, and functioning referral pathways for TB, NCD, and mental health services. A designated programme champion at each site, to troubleshoot workflow barriers, reinforce the supportive purpose of risk stratification, and maintain cascade accountability is a practical necessity. These workforce considerations apply to any organization implementing a risk-stratified model, not only the setting described here. A phased implementation approach may further enhance feasibility, beginning with pilot sites to refine workflows, followed by scale-up supported by mentorship, continuous quality improvement processes, and iterative adaptation to local health system contexts.

## 7 Limitations

This perspective has several limitations. First, CD4 testing is not universally available in all settings, which may constrain formal AHD classification and necessitate reliance on proxy indicators such as clinical staging, treatment interruption, or viral load patterns. Second, EHR data completeness and timeliness vary across facilities, potentially affecting the sensitivity of automated risk triggers and dashboards. Third, the proposed risk-stratification approach is informed by routine programmatic data and regional evidence, but its predictive performance may require local calibration and validation in different epidemiological and health system contexts. In addition, the current Perspective does not directly quantify the duration of key viral non-suppression (VLNS) cascade steps within the TASO cohort, including time from elevated viral load to documented clinical action, repeat testing, or regimen decision-making, nor does it evaluate the independent association between sociodemographic or clinical factors and these time-to-resolution outcomes. However, we draw on external evidence from comparable African settings showing that such delays are common and clinically important, including their association with worse outcomes after confirmed virological failure (3, 4). These represent important priorities for future operational and implementation-focused analyses. Finally, full implementation of the AHD package depends on the availability of diagnostics, medicines, and supply chains particularly for TB, NCDs and opportunistic infection screening which may be uneven across facilities. These constraints underscore the importance of adapting the model pragmatically while maintaining its core principles of timeliness, accountability, and equity.

## 8 Conclusion

Uganda’s rapid transition to DTG and other INSTI-based regimens is a major achievement but does not guarantee durable suppression in the presence of documented delays in the viral load cascade in comparable African settings, structural vulnerability, residual advanced HIV disease, and multimorbidity. Evidence from the TASO cohort and the East African meta-analysis converges: VF is patterned and therefore preventable with service redesign. We propose policy reforms that operationalize a data-enabled, risk-stratified approach that (i) makes the VL cascade a monitored care pathway with time-bound actions, (ii) incorporates a proposed time-limited stability bundle to address adherence, socioeconomic barriers and mental health-informed needs, (iii) integrates TB and NCD care within DSD platforms, and (iv) uses transparent EHR-enabled risk tiering to trigger intensification and de-escalation. The immediate next step is a minimum viable dashboard for programme accountability, followed by iterative refinement of predictive models and evaluation. Crucially, the success of this approach depends not only on data availability, but on the capacity of health systems to translate data into timely, coordinated, and patient-centered action. Implemented with strong data governance, privacy safeguards, and continuous quality improvement, this approach can convert DTG-era scale-up into durable viral suppression while narrowing inequities by sex, age, and socioeconomic status. This Perspective is intended to define a pragmatic implementation framework rather than to present a fully evaluated intervention.

## Data Availability

The data analyzed in this study is subject to the following licenses/restrictions: The data underlying this Perspective are derived from routine programmatic records of The AIDS Support Organisation (TASO) and are not publicly available due to ethical and governance restrictions related to patient confidentiality. Access to de-identified data may be requested from TASO in writing and is subject to review and approval by the TASO Data Safety and Monitoring Board (DSMB), in accordance with institutional and national data governance policies. Requests to access these datasets should be directed to The AIDS Support Organisation (TASO), Uganda | Email: info@tasouganda.org | Website: https://tasouganda.org/.

## References

[1] Dsduganda Implementation-guide-for-differentiated-service-delivery-models-of-hiv-services-in-uganda-20200313.pdf. (2020). Available online at: https://dsduganda.com/wp-content/uploads/2021/08/implementation-guide-for-differentiated-service-delivery-models-of-hiv-services-in-uganda-20200313.pdf (Accessed April 15, 2026).

[2] Namaganda MM Sendagire H Kateete DP Kigozi E Luutu Nsubuga M Ashaba Katabazi F . Next-generation sequencing (NGS) reveals low-abundance HIV-1 drug resistance mutations among patients experiencing virological failure at the time of therapy switching in Uganda. F1000Res. (2022) 11:1–12. doi: 10.12688/f1000research.121980.1

[3] Narainsamy D Mahomed S. Delays in switching patients onto second-line antiretroviral treatment at a public hospital in eThekwini, KwaZulu-Natal. South Afr J HIV Med. (2017) 18:696. doi: 10.4102/sajhivmed.v18i1.696, 29568632 PMC5843064

[4] Bell-Gorrod H Fox MP Boulle A Prozesky H Wood R Tanser F . The impact of delayed switch to second-line antiretroviral therapy on mortality, depending on definition of failure time and CD4 count at failure. Am J Epidemiol. (2020) 189:811–9. doi: 10.1093/aje/kwaa049, 32219384 PMC7523585

[5] Namaganda MM Gennatas S Merson L Garcia E Edinburgh T Nabende JN . Decade-long antiretroviral therapy in Uganda: population-health outcomes from a national HIV treatment cohort, 2014–2024. PLOS Glob Public Health. (2026) 6:e0005906. doi: 10.1371/journal.pgph.0005906, 41838752 PMC12991256

[6] Lamorde M Atwiine M Owarwo NC Ddungu A Laker EO Mubiru F . Dolutegravir-associated hyperglycaemia in patients with HIV. Lancet HIV. (2020) 7:e461–2. doi: 10.1016/S2352-3018(20)30042-4, 32105626

[7] Namaganda MM Mukasa Kafeero H Nakatumba Nabende J Kateete DP Batte C Wanyengera M . Prevalence and predictors of virological failure among the people living with HIV on antiretroviral treatment in East Africa: evidence from a systematic review with meta-analysis and meta-regression of published studies from 2016 to 2023. HIV Res Clin Pract. (2025) 26:2490774. doi: 10.1080/25787489.2025.2490774, 40219653 PMC12182973

[8] TASO Uganda Home – TASO (2017). Available online at: https://tasouganda.org/ (Accessed April 15, 2026).

[9] Zakumumpa H Kitutu FE Ndagije HB Diana NK Ssanyu JN Kiguba R. Provider perspectives on the acceptability and tolerability of dolutegravir-based anti-retroviral therapy after national roll-out in Uganda: a qualitative study. BMC Infect Dis. (2021) 21:1222. doi: 10.1186/s12879-021-06933-8, 34876050 PMC8650263

[10] Buju RT Akilimali PZ Kamangu EN Mesia GK Kayembe JMN Situakibanza HN. Predictors of viral non-suppression among patients living with HIV under Dolutegravir in Bunia, Democratic Republic of Congo: a prospective cohort study. Int J Environ Res Public Health. (2022) 19:1085. doi: 10.3390/ijerph19031085, 35162109 PMC8834045

[11] Cresswell FV Lamorde M. Implementation of long-acting antiretroviral therapy in low-income and middle-income countries. Curr Opin HIV AIDS. (2022) 17:127–34. doi: 10.1097/COH.0000000000000732, 35439787

[12] PrEPWatch Uganda-Consolidated-HIV-and-AIDS-Guidelines-20230516.pdf. (2022). Available online at: https://www.prepwatch.org/wp-content/uploads/2025/11/Uganda-Consolidated-HIV-and-AIDS-Guidelines-20230516.pdf (Accessed April 15, 2026).

[13] Mojola SA Angotti N Denardo D Schatz E Xavier Gómez Olivé F. The end of aids? HIV and the new landscape of illness in rural South Africa. Glob Public Health. (2022) 17:13–25. doi: 10.1080/17441692.2020.1851743, 33290168 PMC8184878

[14] Kiragga AN Mubiru F Kambugu AD Kamya MR Castelnuovo B. A decade of antiretroviral therapy in Uganda: what are the emerging causes of death? BMC Infect Dis. (2019) 19:77. doi: 10.1186/s12879-019-3724-x, 30665434 PMC6341568

[15] Amuri M Mitchell S Cockcroft A Andersson N. Socio-economic status and HIV/AIDS stigma in Tanzania. AIDS Care. (2011) 23:378–82. doi: 10.1080/09540121.2010.507739, 21347901

[16] Igulot P Magadi MA. Socioeconomic status and vulnerability to HIV infection in Uganda: evidence from multilevel modelling of AIDS indicator survey data. AIDS Res Treat. (2018) 2018:7812146. doi: 10.1155/2018/7812146, 29983999 PMC6011175

[17] Plymoth M Sanders EJ Van Der Elst EM Medstrand P Tesfaye F Winqvist N . Socio-economic condition and lack of virological suppression among adults and adolescents receiving antiretroviral therapy in Ethiopia. PLoS One. (2020) 15:e0244066. doi: 10.1371/journal.pone.0244066, 33320900 PMC7737988

[18] UNAIDS Global HIV & AIDS statistics — Fact sheet. (2025). Available online at: https://www.unaids.org/en/resources/fact-sheet (Accessed April 15, 2026).

